# Lower Lip Reconstruction Using the Karapandzic Flap Technique

**DOI:** 10.7759/cureus.50929

**Published:** 2023-12-21

**Authors:** Rui Suzuki, Nayon Kimura

**Affiliations:** 1 Department of Plastic and Reconstructive Surgery, Beppu Medical Center, National Hospital Organization (NHO), Beppu, JPN

**Keywords:** perioral structures, basal cell carcinoma, skin cancer, lower lip reconstruction, karapandzic flap

## Abstract

The perioral region, comprising the upper and lower lips, plays important functional, aesthetic, and anatomical roles. Postoperative defects in perioral structures present a considerable challenge for reconstruction. Currently, reconstruction of perioral structures is performed using local, distant, and free flaps. Herein, we present a case of reconstruction with a Karapandzic flap after the excision of basal cell carcinoma in the lower lip. The patient was a 76-year-old man with a skin tumor on his left lower lip. He consulted a dermatologist regarding the tumor growth and was diagnosed with basal cell carcinoma upon biopsy. Dermatological excision of the tumor at a 7-mm margin resulted in a defect in half of the lower lip, cheek skin, and corner of the mouth. The defects were reconstructed using a Karapandzic flap for functional considerations. The patient was satisfied with his aesthetic appearance, and no functional deficits were observed. In conclusion, the Karapandzic flap is suitable for reconstructing large defects of the lower lip and can be completed quickly and safely in a single procedure.

## Introduction

Reconstruction of defects in the lower lip is surgically challenging. Malignant lesions that involve the angle of the mouth and lower lip warrant wide excision to ensure disease-free margins. The resulting defects are usually large and often involve half of the lower lip. Free, distant, and local flaps are available for the reconstruction of such defects. However, distant and free flaps are usually bulky, require a secondary procedure, seldom match the color of the facial skin, and often fail to establish a proper vermilion border and lip competence. An ideal reconstruction technique should comprise a single-stage procedure that reliably replaces the defect with similar tissue and restores aesthetics and function [1]. Several techniques have been suggested for the reconstruction of peroral defects, including methods by Abbe, Estlander, Bernard, Zisser, Gillies, and Karapandzic [2-8].

In 1974, Karapandzic [4] described the use of a neurovascular myocutaneous flap with satisfactory outcomes. The Karapandzic flap has distinct advantages; it allows functional preservation of the orbicularis oris muscle, facial arteries, and sensory and motor nerves, resulting in the retention of sensory functions, a mobile lip, and improved lip capacity [4]. Herein, we present a case of reconstruction with a Karapandzic flap after the excision of basal cell carcinoma in the lower lip.

## Case presentation

**Figure 1:**
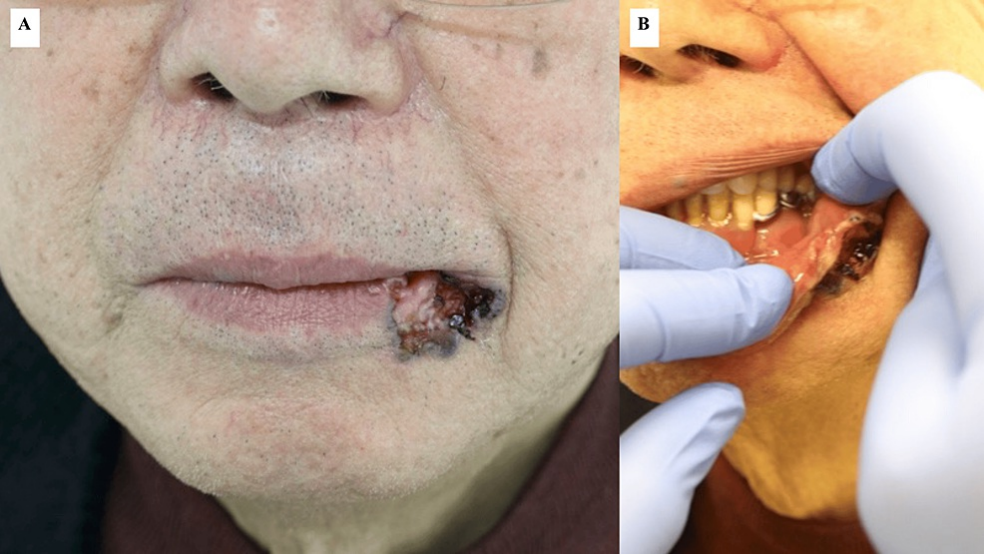
Clinical findings. (A) The lesion is located on the left side of the lower lip, measuring 23 mm x 22 mm, including the corner of the mouth, with an ulcer in the center and a black plaque at the margin. Induration is observed throughout the area immediately below. (B) The lesion involves the lower lip mucosa, red lip border, and cheek skin.

**Figure 2:**
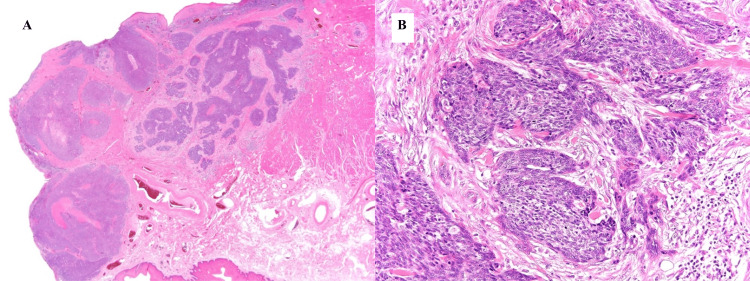
Pathological findings. (A) The Loupe image. In some areas, basaloid cells are observed to proliferate and form nests of various sizes in continuation with the normal epidermis. (B) A high-power view. Palisading is observed around the nest, and cleft formation is observed between the nests, which are findings of basal cell carcinoma.

A 76-year-old man was referred to the dermatology department because of a skin tumor on the left corner of his mouth that had gradually increased in size over the past 11 years. The patient had a history of diabetes, hypertension, and cerebral infarction. He was previously hesitant to seek treatment; however, the tumor had become ulcerated and difficult to manage. The lesion was on the left side of the lower lip, 23 mm × 22 mm in size, and included the corners of the mouth. Additionally, the lesion had ulceration in the center, black patches on the edges, and induration over the entire area immediately below the ulceration, which involved the mucous membrane, vermilion border, and skin of the lower lip (Figure 1). Based on a preoperative skin biopsy, the patient was diagnosed with basal cell carcinoma (Figure 2).

**Figure 3:**
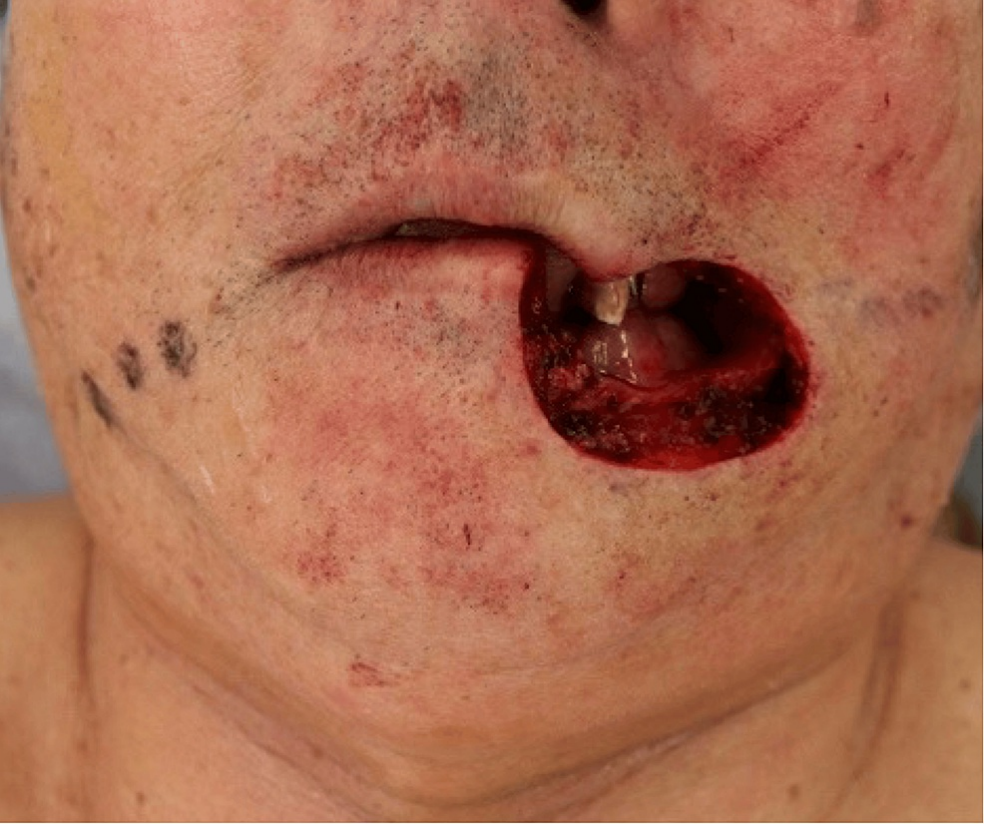
After lesion removal. Image of the area immediately after lesion removal by the dermatologist. The defect size is 45 mm × 44 mm.

**Figure 4:**
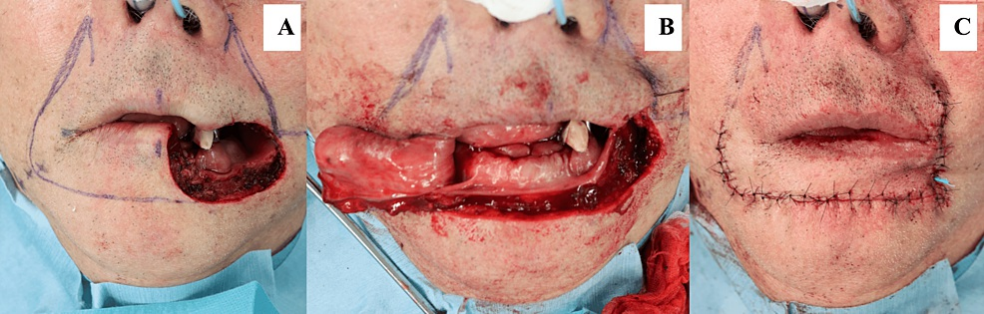
Karapandzic flap procedure. (A) A Karapandzic flap is designed from the mentolabial sulcus to the nasolabial sulcus. (B) After skin flap elevation. The entire thickness of the lower lip is elevated. (C) After flap reconstruction.

The dermatologist planned extensive excision of the lesion, and the tumor was excised with a safety margin of 7 mm. This resulted in a large defect involving approximately half of the lower lip, cheek skin, and corners of the mouth. Owing to the size of the defect, primary closure was not feasible, and the use of a Karapandzic flap was planned (Figure 3). A Karapandzic flap was designed along the right nasolabial sulcus from the mentolabial sulcus, and a left auxiliary incision was designed to fit the nasolabial sulcus. The oral vestibule was elevated using a mental region flap in full layers, and the buccal area was elevated using a skin valve on the superficial musculoaponeurotic system (SMAS). The right facial artery was not dissected because its width was sufficient without requiring exposure of the artery. The auxiliary incision line on the left side allowed coverage of the defect without additional incisions (Figure 4).

**Figure 5:**
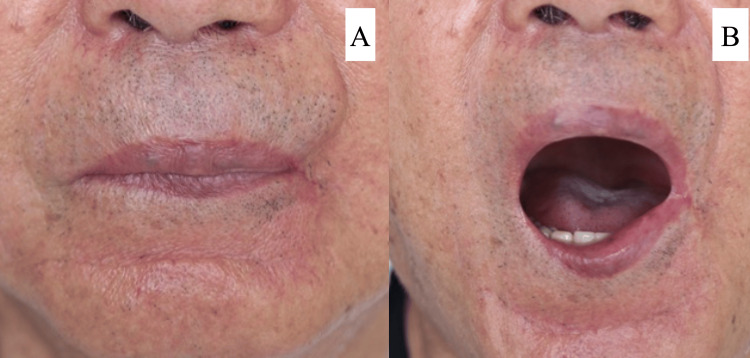
Post-surgery (A) The findings six months postoperatively reveal even red lip margins, and the scar at the skin flap collection site is not noticeable. (B) Although thinning of the red lip mucosa of the lower lip is observed, the third lateral finger can be opened, and cosmetic satisfaction is high.

At six months postoperatively, the patient showed no evidence of tumor recurrence. Although there was thinning of the red-lipped mucosa of the lower lip in terms of cosmetic appearance, the two lateral fingers were able to open. The patient was satisfied with the results and reported no functional limitations (Figure 5).

## Discussion

Treatment of cancer in the perioral region requires resection according to the oncological principles and consideration of functional and aesthetic results. Nevertheless, disease-free resection is preferred to maintain function and restore aesthetics.

An optimal method for lip reconstruction is yet to be established. In the lower lip, if the lesion involves less than one-third of the lip, direct closure can be performed; otherwise, valvuloplasty may be performed. The lip tissue consists of the skin, muscle layer, red lips, and oral mucosa and has a characteristic structure that is difficult to find in other parts of the body. Therefore, using nearby lip tissues for lower lip reconstruction is suitable. As previously mentioned, if the lesion covers more than 35% of the lip, flap reconstruction is necessary, and the consideration of adjacent areas, such as the unaffected lip (Abbe type), cheek (Gilles or Estlander type), and chin (Bernard type), are required. 

The Karapandzic procedure [4] is advantageous because it requires only a single surgery, can be performed quickly once learned, preserves important vascular and nerve networks, is safe, and maintains lip mobility and sensation. However, the disadvantages include asymmetry of the corners of the mouth and a reduction in the overall size of the mouth. Regarding the asymmetry of the corners of the mouth, Jackson [9] stated that a potential problem regarding the size of the mouth is not a critical issue. Asymmetry of the corners of the mouth improves over time, and Jackson recommended the Karapandzic method over other methods of lower lip reconstruction that result in stiff, cold, and nonfunctional lips [9]. In this case, the most important aspect of reconstruction was the functional reconstruction of the corner of the mouth. At the corner of the mouth, the orbicularis oris muscle is composed of a complex structure that includes the risorius, levator anguli oris, buccinator muscle, and depressor anguli oris muscles. Therefore, herein, we believe that using the orbicularis oris muscle, which has sufficient muscle mass, to reconstruct the corner of the mouth functionally was the most favorable option. Additionally, the Abbe technique is unsuitable for the corners of the mouth. With these considerations, the Karapandzic flap technique was deemed the most suitable in the present case. Because the normal contralateral corner of the mouth remains, this is the subject of comparison. However, irrespective of the selected lip reconstruction method, asymmetry of the corners of the mouth will occur. If emphasis is placed on the shape of the mouth, insufficient muscle reconstruction can result in dispersion and lead to numbness in the corners of the mouth, thereby reducing the significance of reconstruction. In our case, the corners of the mouth were maintained in a good position, and the course was good with no complications such as castration.

## Conclusions

The Karapandzic flap is suitable for reconstructing large defects of the lower lip, including the corners of the mouth, as it can be completed quickly and safely in a single procedure. In addition, this technique allows for neurovascular pedicle preservation and provides good functional and aesthetic outcomes.

However, this technique has the limitation of potential microstomia during the early stages. Despite this, we believe that this is a good option for flap reconstruction of lips because the resulting microstomia improves over time.
